# Extracorporeal life support in severe drug intoxication: a retrospective cohort study of seventeen cases

**DOI:** 10.1186/cc8017

**Published:** 2009-08-25

**Authors:** Cédric Daubin, Philippe Lehoux, Calin Ivascau, Marine Tasle, Mehdi Bousta, Olivier Lepage, Charlotte Quentin, Massimo Massetti, Pierre Charbonneau

**Affiliations:** 1 Department of Medical Intensive Care, Caen University Hospital, avenue Côte de Nacre, Caen Cedex 14033, France; 2 Department of Anesthesiology, Caen University Hospital, avenue Côte de Nacre, Caen Cedex 14033, France; 3 Department of Thoracic and Cardiovascular Surgery, Caen University Hospital, avenue Côte de Nacre, Caen Cedex 14033, France

## Abstract

**Introduction:**

Cardiovascular failure is the leading cause of death in severe acute drug intoxication. In this setting, we report the feasibility, complications, and outcome of emergency extracorporeal life support (ECLS) in refractory shock or cardiac arrest following a drug overdose.

**Methods:**

This is a retrospective cohort study of 17 patients admitted over a 10-year period for prolonged cardiac arrest or refractory shock following a drug overdose and not responding to optimal conventional treatment. Patients were evaluated in the medical ICU and cardiovascular surgery department of a university hospital. ECLS implantation used a centrifugal pump connected to a hollow-fiber membrane oxygenator and was performed in the operating room (n = 13), intensive care unit (n = 3), or emergency department (n = 1). ECLS was employed for refractory shock and prolonged cardiac arrest in 10 and 7 cases, respectively.

**Results:**

The mean duration of external cardiac massage was 101 ± 55 minutes. Fifteen patients had ingested cardiotoxic drugs, including 11 cases of drugs with membrane stabilizing activity. Time from hospital admission to initiation of ECLS was 6.4 ± 7.0 hours. Time to ECLS implant was 58 ± 11 minutes. The mean ECLS flow rate was 3.45 ± 0.45 L/min. The average ECLS duration was 4.5 ± 2.4 days. Early complications included limb ischemia (n = 6), femoral thrombus (n = 1), cava inferior thrombus (n = 1), and severe bleeding at the site of cannulation (n = 2). Fifteen patients were weaned off ECLS support and 13 (76%) were discharged to hospital without sequelae.

**Conclusions:**

Based on our experience, we consider ECLS as a last resort, efficient, and relatively safe therapeutic option in this population. However, the uncontrolled nature of our data requires careful interpretation.

## Introduction

Drug-induced cardiovascular failure is the leading cause of death in severe acute drug intoxication [1,2]. In this setting, patients with refractory shock or cardiac arrest who do not respond to optimal conventional treatment may need special therapies, such as extracorporeal life support (ECLS). Although cardiovascular bypass is rarely used in the management of poisoning, it may have potential benefits for hemodynamic instability not responding to conventional measures. Promising results have been obtained using temporary circulatory support in several single-case reports [3-17] and short series [18,19]. However, the usefulness of cardiovascular bypass in drug-induced cardiac failure remains unclear [20]. The aim of the study was to describe our 10-year experience of ECLS as a last resort therapeutic option in acute poisoning. Seven of the cases included in this series were previously published [15,18].

## Materials and methods

We reviewed the cases of all patients treated with emergency cardiopulmonary bypass for prolonged cardiac arrest or cardiogenic shock following drug intoxication at the University Hospital of Caen between 1997 and 2007. Our medical teams and nurses have a large amount of experience with emergency ECLS, specifically among critically ill patients [15,18,21,22].

### Patients

**Figure 1:**
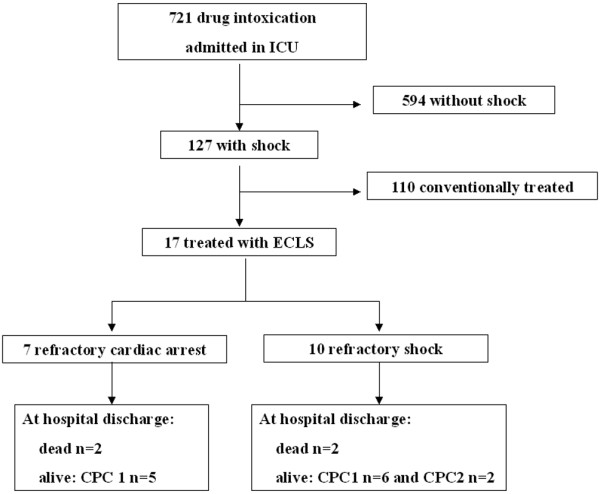
Flow chart indicating patient characteristics. CPC: cerebral performance class; ECLS: extra corporeal life support; ICU: intensive care unit.

During the study period, 721 patients were admitted for drug intoxication (Figure 1). One hundred and ten patients had hemodynamic failure responding to conventional treatment and 17 patients had refractory shock or cardiac arrest. In our practice, patients with refractory cardiac arrest, defined as an absence of return to spontaneous circulation after continuous cardio-pulmonary resuscitation over at least 45 minutes or refractory shock, defined as shock not responding to optimal conventional treatment, were candidates for ECLS support [23]. When the decision to implant ECLS was made by a senior intensivist, a senior cardiac surgeon and a perfusionist were immediately informed and ECLS performed.

### Data collection

According to French legislation at the time of the study and given the observational retrospective nature of the study, no ethical committee was requested and thus no informed consent was obtained from the patients. At the time of ECLS implantation the focus was specifically on hemodynamic (including electrocardiographic and echocardiographic), neurologic, respiratory, renal, liver, and hematologic data. Measured physiological variables were used to calculate the Simplified Acute Physiology Score (SAPS II) [24] and the Sequential Organ Failure Assessment (SOFA) score [25]. The toxicological screening was recorded and drugs classified as with or without cardiotoxic effect or membrane stabilizing activity (MSA). In addition, the clinical course of each patient during hospitalization was recorded. Vascular, neurologic, hemorrhagic, renal, and perfusion system complications were documented. The neurologic outcome at hospital discharge was assessed according to the cerebral performance class (CPC) categories [26]: CPC 1 = good cerebral performance, CPC 2 = moderate cerebral disability, CPC 3 = severe cerebral disability, CPC 4 = coma or vegetative state, and CPC 5 = brain death or death.

### Cannulation technique

Device description, cannulation technique, management, and weaning from ECLS were previously reported in detail [22]. Briefly, the hardware for cardiopulmonary circulation consisted of a Biomedicus portable system (Medtronic, Inc, Minneapolis, MN, USA) incorporating a centrifugal pump console and a water pump system. The closed ECLS circuit consisted of pre-connected polyvinyl chloride tubing (Medtronic, Inc, Minneapolis, MN, USA) including a constrained vortex pump chamber, a hollow-fiber membrane oxygenator with an integral heat exchanger (Maxima PRF, Medtronic, Inc, Minneapolis, MN, USA), and a flow probe. All components were heparin-coated (Carmeda Bioactive Surface-coating). The cannulae were Biomedicus (17 F to 25 F), according to the size of patients.

Once the decision to implant ECLS support was made, the circuit was quickly primed with normal saline. Heparin was administered to the patient at 50 UI/kg immediately before cannulation of the vessels. The activated clotting time (ACT) was kept between 150 and 200 seconds at full-flow assistance. Peripheral femorofemoral cannulation was surgically set up using a modified Seldinger technique. Because cannulation-related limb ischemia was a major problem when we began this technique, additional distal limb perfusion was inserted to avoid severe leg ischemia. The distal tip of the arterial cannula was positioned in the common iliac artery or distal abdominal aorta, whereas the tip of the venous cannula was set in the right atrium under echocardiographic guidance and confirmed by chest radiograph.

### Patient management during ECLS

Pump flow was initially set at 2.5 L/m^2 ^and vasopressor (norepinephrine and epinephrine) was used to maintain a mean blood pressure of at least 60 mmHg. An inotropic support was used even if systemic perfusion was adequately performed by ECLS to maintain a pulsatile flow through the native heart. The aim was to decompress the left heart and minimize stasis, therefore reducing the risk of intracardiac thrombosis. If necessary, to accomplish mechanical decompression of the left heart, an atrial balloon septostomy was performed. A femoral vein approach was used in which a transeptal puncture, followed by blade septostomy, was performed under combined radioscopy and echocardiographic guidance. Sequential balloon inflations were carried out to achieve left heart decompression, which was confirmed by echocardiography. The ECLS was monitored by trained ICU personnel. A perfusionist was also available for occasional monitoring visits and emergencies. Echocardiography was used serially to assess progressive myocardial recovery and exclude intracardiac thrombosis or other abnormalities. All patients were mechanically ventilated with 5 to 6 mL/kg tidal volume and 8 to 10 cmH_2_O positive end-expiratory pressure, and continuous venovenous hemofiltration was used to treat acute renal failure and regulate the intravascular volume and overall fluid balance, if necessary.

### Weaning

The decision to discontinue ECLS support was based on evidence of multiorgan failure, overwhelming sepsis, or severe neurological injury. Patients were weaned off ECLS if the left ventricular ejection fraction, assessed by echocardiography during a reduction of pump flow to 500 to 1000 mL/min, was stable (> 50%) without deterioration in hemodynamic status. During this period, anticoagulation was adapted to adequate values of ACT (250 to 300 seconds). If the patient's cardiovascular status remained stable, ECLS was withdrawn by cardiac surgeons.

### Statistical analysis

Quantitative and qualitative data are expressed as mean (± standard deviation), or median (range) and percentage, respectively.

## Results

### Patients and drugs

Seventeen patients (11 females, 6 males; mean age 39 ± 18 years) were treated with ECLS following drug intoxication. All patients, except two, had ingested cardiotoxic drugs, including 11 cases of drugs with MSA. The majority (12/17) of poisonings resulted from mixed poisonings involving a combination of cardiotoxic drugs, various psychotic drugs, and alcohol (Table 1).

**Table 1:** Patients and drugs used

Patients	Drugs
**1**	Sotalol 4.8 g, verapamil 7.2 g
**2**	Disopyramide* 10 g, alprazolam 10 mg
**3**	Acebutolol* 10 g, méprobamate 4 g, aspirin 15 g, alprazolam 10 mg
**4**	Tianeptine, Bromazepam, fluoxetine, zolpidem 4 g
**5**	Verapamil 1.2 g, propanolol* 4 g, betaxolol 6.8 g
**6**	Acebutolol* 8 g, meprobamate 5 g
**7**	Flecaine*8 g, venlafaxine
**8**	Verapamil
**9**	Disopyramide* 3 g, lithium 7.5 g, citalopram 0.2 g
**10**	Propanolol* 2 g, meprobamate, paroxetine, paracetamol, dextropropoxyfene
**11**	Metoprolol, alcohol
**12**	Verapamil 3.6 g
**13**	Propanolol* 2 g, alcohol
**14**	Cibenzoline*, benzodiazepines
**15**	Tramadol 10 g, hydroxyzine 6 g, gabapertine 1 g, clonazepam 80 mg
**16**	Propafénone*, alcohol, oxilamine
**17**	Propanolol*

* Drugs with membrane stabilizing activity (MSA).

### Baseline characteristics

The baseline characteristics of patients at the time of ECLS implantation are reported in Table 2. All patients, except two, were comatose. The patients' median SAPS II score was 69 (26 to 82) and median SOFA score was 13.5 (3 to 18). The ECLS setup was performed for 13 patients in the operating room, 3 in the intensive care unit, and 1 in the emergency department. Seven patients received ECLS during external cardiac massage for refractory cardiac arrest, which occurred in six cases at hospital admissions in the emergency room. Ten other patients also received ECLS, including two after the restoration of spontaneous circulation following a brief period of asystole, during refractory shock. The mean duration of external cardiac massage was 101 ± 55 minutes.

**Table 2:** Baseline characteristics at the time of ECLS implantation*

N°	ECM (min)	GCS	HR	AP (mmHg)	Vasopressor	ECG	LVEF	Lactate (mmol/L)	Bicarbonate (mmol/L)
**1^§^**	160	3	0	0	Epinephrine	Asystole	Akinesia	-	21.1
**2^§¶^**	150	3	0	0	Epinephrine	Asystole	-	> 30	17.8
**3^§¶^**	170	3	0	0	Epinephrine Isoproterenol	Asystole	Akinesia	10.8	19
**4**	60	3	40	60/40	Epinephrine	Bradycardia (QRS 183 ms)	Hypokinesia (LEVF 15%)	5.85	29.6
**5**	70	3	0	0	Epinephrine	Asystole	-	7.3	26.3
**6**	50	3	0	0	-	Asystole	Akinesia	-	-
**7**	60	3	30	50/33	Epinephrine Dobutamine	Bradycardia (QRS 214 ms)	Akinesia	-	17.5
**8^¶^**	No	3	60	50/30	Epinephrine Isoproterenol	Sinusal	-	10.5	17.7
**9**	No	3	40	85/54	Epinephrine Isoproterenol Dobutamine	Bradycardia (QRS 175 ms)	Hypokinesia	9.65	19.6
**10^¶^**	No	3	68	71/52	Epinephrine Dobutamine	Sinusal	Hypokinesia (LVEF 20%)	0.57	17.2
**11**	No	3		60/48	Epinephrine Dobutamine	Sinusal	Hypokinesia (LVEF 20%)	-	-
**12**	No	8	30	70/40	Norepinephrine Isoproterenol Dopamine	Atrio-ventricular block	Hypokinesia (LVEF 25%)	4.6	17.9
13**	No	3	55	75/50	Epinephrine	Sinusal	Hypokinesia (LVEF 30%)	5.9	11.7
**14^§¶^**	No	3	140	85/58	Epinephrine	Ventricular tachycardia	Hypokinesia (LVEF 20%)	8,8	20.2
15**^¶^	No	3	85	82/56	Epinephrine Norepinephrine	Right bundle-branch block	Hypokinesia (LVEF 25%)	1.7	18
**16**	No	15	60	70/60	Epinephrine	Bradycardia (QRS 203 ms)	Hypokinesia (LVEF 10%)	3.7	22.4
**17**	No	15	36	60/40	Epinephrine Dobutamine	Sinusal	Hypokinesia (LVEF 10%)	3.7	19.9

* For patients with cardiac arrest, we provided data recorded just before cardiac arrest if available

** ECLS performed after restoration of spontaneous circulation following a brief period of asystole

^§ ^Patients with temporary external transthoracic electrostimulation before initiation of ECLS

^¶ ^Patients requiring continuous venovenous hemofiltration or conventional dialysis before or immediately following ECLS implantation

AP: arterial pressure; ECG: echocardiogram; ECM: manual external cardiac massage; ECLS: extra corporeal life support; HR: heart rate; GCS: Glasgow coma score; LVEF: left ventricular ejection fraction; QRS: duration of the complex representing ventricular depolarization on electrocardiogram.

Before the initiation of ECLS support, a severe decrease in cardiac contractility was documented by echocardiography in 14 cases. All patients were mechanically ventilated and received vasopressor. Four patients needed temporary external transthoracic electrostimulation. Six patients required continuous venovenous hemofiltration or conventional dialysis for acute renal failure before or immediately after ECLS implantation. Before connection to ECLS, the median arterial pH was 7.37 (7.34 to 7.41), partial presure of arterial oxygen/fraction of inspired oxygen ratio was 239 (180 to 261), serum bicarbonate concentration was 19.0 mmol/L (17.7 to 20.6), plasma lactate concentration was 5.9 mmol/L (3.7 to 9.7), and serum creatinine concentration was 160 μmol/L (114 to 204).

### ECLS feasibility

ECLS feasibility, assessed with respect to time from admission to ECLS initiation, and the percentage of successful procedures (i.e. flow rate > 2.5 L/m^2 ^and mean blood pressure > 60 mmHg) is shown in Table 3. Time from hospital admission to initiation of ECLS was 6.4 ± 7.0 hours, and the time to ECLS implant was 58 ± 11 minutes. The mean ECLS flow rate was 3.45 ± 0.45 L/min. The average ECLS duration was 4.5 ± 2.4 days. In one patient (no. 17), an atrial balloon septostomy was performed to accomplish mechanical decompression of the left heart.

**Table 3:** ECLS feasibility, duration and complications

N°	Time from admission to initiation ECLS (hours)	Time to implant ECLS* (min)	Initial ECLS flow rate (l/min)	ECLS duration (days)	LVEF at ECLS discharge	Long term surviving
1**	2.5	60	3.5	2	-	No
2**	1.5	60	3	3	-	No
**3**	2 hours 50 mins	40	3.5	2.3	62%	Yes
**4**	1	60	3.5	2.5	> 50%	Yes
**5**	1	60	-	2.5	76%	Yes
**6**	1	60	3.5	2.3	-	Yes
**7**	6	60	3.6	3	56%	Yes
**8**	5	60	3.7	11	-	No
**9**	6	60	3.4	5	normal	No
**10**	24	60	4	5	42%	Yes
**11**	4	60	-	4	38%	Yes
**12**	2	60	4	6	> 50%	Yes
**13**	2.5	45	2.6	7	74%	Yes
**14**	15	90	4	6	45%	Yes
**15**	16	60	2.5	8	45%	Yes
**16**	3	60	3.5	4	50%	Yes
**17**	17	40	3.5	3	40%	Yes

* Delay between ECLS decision and time at which ECLS flow was provided

** Patients dead during ECLS assistance

ECLS: extra corporeal life support; LVEF: left ventricular ejection fraction.

### ECLS complications

Significant cannulation-related injuries of femoral vessels were reported in 10 patients: six patients with limb ischemia requiring urgent revascularization in three cases, one femoral thrombus, one cava inferior thrombus, and two cases of severe bleeding at the site of cannulation requiring a surgical revision.

### Clinical outcome

Fifteen patients were weaned off ECLS support and two patients withdrawn from support because of refractory multiorgan failure and cerebral death (Table 3). Thirteen patients survived and were discharged to hospital without significant cardiovascular or neurological sequelae (CPC 1 n = 9 and CPC 2 n = 4). Two patients died of septic shock and cerebral death during the hospital stay.

## Discussion

We report one of the largest series of drug-induced cardiac arrest and refractory shock managed with ECLS. The high survival rate (76%) reported in this setting supports ECLS as an efficient rescue treatment in a subset of patients with drug-induced circulatory failure not responding to optimal conventional treatment.

### Patients and drugs

Clinical experience with emergency ECLS during poisoning leading to prolonged cardiac arrest or shock that does not respond to conventional treatment is limited [3-19]. Poly-intoxication including cardiotoxic drugs with MSA, which is known to be associated with a high mortality rate [27], was involved in a majority of cases, as previously reported [28]. All patients were in prolonged cardiac arrest or refractory shock according to the definitions proposed by Baud and colleagues [28].

### ECLS feasibility and efficiency

We confirmed the feasibility of emergency ECLS in accordance with previous reports focusing on prolonged cardiac arrest regardless of the cause [19,22]. The physiologic objective was to provide temporal circulatory support to the vital organs and unload the failing heart as the injured myocardium attempts to recover. In previous cohort studies [19,22], survival rates were clearly higher in the toxic cardiac arrest group, as compared with other causes of cardiac arrest (3 of 12 vs 0 of 5 [19] and 4 of 6 vs 4 of 34 [22], respectively). The high survival rate (76%) reported in our cohort was in accordance with the general survival rate from 58% (15 of 26) in case reports of poisoned patients who benefited from ECLS [3-14,16,17,19]. The 5 of 7 (71%) survival rate we reported among patients with cardiac arrest was in contrast with the dramatically low survival rate of 7% and 4.5%, respectively, reported in overdoses involving cardiac arrest [29,30]. To our knowledge, no studies have reported the survival rate of patients with drug-induced cardiovascular shock apparently refractory to conventional treatment, limiting comparisons with our cohort. However, experimental studies with control groups demonstrated that ECLS improved survival in animal models of severe cardiotoxic drug-induced shock [31,32]. These results suggest that ECLS could be considered as a good emergency resuscitative tool in this setting.

### ECLS complications

Severe cannulation-related limb ischemia was the major problem when we first started the technique. Therefore, to accomplish a perfusion of the distal limb, surgeries performed an additional arterial shunt with a small 8 F catheter between the side port of arterial cannula and a point located some centimeters distally in the superficial femoral artery shunt. After this supplementary shunt was added, only distal embolic ischemia were reported. In contrast to Mégarbane and colleagues [19], the rate of cannulation-related complications, limb ischemia, and major bleeding was relatively high despite the modified Seldinger technique and additional distal limb perfusion. These differences could be explained by a higher ECLS duration in our study. However, our results were in accordance with reports of significant morbidity associated with emergency ECLS [33]. In addition, no death was induced by cannulation-related complications, and all survivors were discharged without significant cardiovascular or neurological sequelae.

### Limitations

Firstly, because ECLS indication for drug overdose is rare, the sample size is small. Secondly, the uncontrolled retrospective observational design does not permit clarification of the role of ECLS therapy in drug-induced cardiac failure in a therapeutic algorithm. However, because usefulness of ECLS in this setting remains debatable [20], we believe this study adds important information about ECLS as a rescue therapy in patients with drug-induced cardiac arrest and refractory shock.

## Conclusions

Based on our experience, we consider ECLS as a last resort, efficient, and relatively safe therapeutic option in critically ill poisoned patients (i.e. cardiac arrest and refractory shock) who do not respond to conventional therapies, providing the cardiac surgeon with the means to rapidly intervene and control ECLS-related complications. However, because there is insufficient evidence concerning the use of ECLS as a treatment for severe cardiac impairment due to poisoning, further studies are needed to clarify criteria for unresponsiveness to conventional treatment and the indications of ECLS in this setting.

## Key messages

• Cardiovascular failure is the leading cause of death following a cardiotoxic drug overdose.

• This report supports the hypothesis that ECLS may be considered as a last resort, efficient, and relatively safe therapeutic option in critically ill poisoned patients (i.e. cardiac arrest and refractory shock) who do not respond to conventional therapies.

## Abbreviations

ACT: activated clotting time; CPC: cerebral performance class; ECLS: extra corporeal life support; MSA: membrane stabilizing activity; SAPS: Simplified Acute Physiology Score; SOFA: Sequential Organ Failure Assessment.

## Competing interests

The authors declare that they have no competing interests.

## Authors' contributions

CD and MB initiated the study, and the design. CD and PC were involved in the interpretation of the results. CD wrote the manuscript, and PC helped to draft the manuscript. PL, CI, MT, OL, MB, CQ, MM, and PC contributed to the conception of the study and revision of the manuscript. All authors read and approved the final manuscript.

## Authors' information

The work has been presented in part at the annual congress of the Société de Réanimation de Langue Française (SRLF) held in January 2008, Paris, France.
